# Small Area Estimation of Cancer Risk Factors and Screening Behaviors in US Counties by Combining Two Large National Health Surveys

**DOI:** 10.5888/pcd16.190013

**Published:** 2019-08-29

**Authors:** Benmei Liu, Van Parsons, Eric J. Feuer, Qiang Pan, Machell Town, Trivellore E. Raghunathan, Nathaniel Schenker, Dawei Xie

**Affiliations:** 1 Division of Cancer Control and Population Sciences, National Cancer Institute, Bethesda, Maryland; 2 National Center for Health Statistics, Centers for Disease Control and Prevention, Hyattsville, Maryland; 3 Harbin Institute of Technology, Shenzhen, Guangdong, China; 4 Division of Population Health, Centers for Disease Control and Prevention, Atlanta, Georgia; 5 Department of Biostatistics and Institute for Social Research, University of Michigan, Ann Arbor, Michigan; 6 National Center for Health Statistics (retired), Centers for Disease Control and Prevention, Hyattsville, Maryland; 7 Department of Biostatistics, Epidemiology, and Informatics, Perelman School of Medicine, University of Pennsylvania, Philadelphia, Pennsylvania

## Abstract

**Background:**

National health surveys, such as the National Health Interview Survey (NHIS) and the Behavioral Risk Factor Surveillance System (BRFSS), collect data on cancer screening and smoking-related measures in the US noninstitutionalized population. These surveys are designed to produce reliable estimates at the national and state levels. However, county-level data are often needed for cancer surveillance and related research.

**Methods:**

To use the large sample sizes of BRFSS and the high response rates and better coverage of NHIS, we applied multilevel models that combined information from both surveys. We also used relevant sources such as census and administrative records. By using these methods, we generated estimates for several cancer risk factors and screening behaviors that are more precise than design-based estimates.

**Results:**

We produced reliable, modeled estimates for 11 outcomes related to smoking and to screening for female breast cancer, cervical cancer, and colorectal cancer. The estimates were produced for 3,112 counties in the United States for the data period from 2008 through 2010.

**Conclusion:**

The modeled estimates corrected for potential noncoverage bias and nonresponse bias in the BRFSS and reduced the variability in NHIS estimates that is attributable to small sample size. The small area estimates produced in this study can serve as a useful resource to the cancer surveillance community.

**Summary**

**What is already known about this topic?**

Data on cancer screening behaviors and risk factors for small geographic areas (eg, counties) are difficult to obtain.

**What is added by this report?**

We used the data from the National Health Interview Survey and the Behavioral Risk Factor Surveillance System to develop a novel statistical method for combining the 2 surveys to produce modeled estimates for 3,112 US counties for 11 outcomes pertaining to smoking and screening for female breast cancer, cervical cancer, and colorectal cancer.

**What are the implications for public health practice?**

Small area estimates can help the cancer surveillance community fulfill multiple needs and goals.

## Introduction

Cancer screening and risk factor data at the state and county levels are useful to cancer control planners, policy makers, and researchers for local health planning, decision making, and resource allocation. However, accurate screening and risk factor data are difficult to obtain. Cost and resource constraints make it impossible to conduct a new study for every new problem of interest.

Large national health surveys (eg, National Health Interview Survey [NHIS], Behavioral Risk Factor Surveillance System [BRFSS]) collect data on cancer screening behaviors and risk factors such as smoking and alcohol intake. NHIS, conducted by the Centers for Disease Control and Prevention’s (CDC’s) National Center for Health Statistics (NCHS) since 1957, is designed to provide estimates by nation, region, and selected states with large populations. BRFSS, conducted by CDC since 1984, is designed to provide estimates for states and substate areas, such as metropolitan and micropolitan statistical areas. However, BRFSS’s sample size is not large enough to provide reliable estimates for relatively small geographic areas (eg, county, state legislative district). Because stand-alone surveys may not support reliable estimation at these lower geographic levels, model-based small area estimates (SAEs) that combine information from multiple sources could be an effective alternative approach.

SAE techniques have a long history (1). Research studies using advanced methods and SAE techniques have been reported in the public health literature (2–5). Many of those studies used a single survey (either NHIS or BRFSS, but not both) as the data source for outcomes. In our study, we aimed to harness the strengths of both NHIS and BRFSS. We developed a novel statistical method to combine the 2 surveys to produce SAEs for smoking prevalence and cancer screening rates for years 1997–1999 and 2000–2003 (6). We extended this approach (6) to incorporate a cellular telephone–only component for data collected in years 2004–2010. We grouped multiple years of data into 2 periods, 2004–2007 and 2008–2010, so that each data period includes 1 or 2 years of NHIS data. This time period grouping enlarges sample sizes for counties with very small or no samples and by using a small time period, avoids smoothing out significant temporal trend changes in outcomes of interest. We describe the methodology used for data period 2008–2010, the most recent data period for which final estimates were calculated.

## Methods

### Data sources

We used 2 major data sources, NHIS 2008–2010 and BRFSS 2008–2010, to obtain estimates for 11 outcomes of interest: 1) current smokers overall, 2) current male smokers, 3) current female smokers, 4) overall ever smokers, 5) male ever smokers, 6) female ever smokers, 7) mammography screening, 8) Papanicolaou test, 9) home fecal occult blood test (HFOBT), 10) colorectal endoscopy, and 11) colorectal cancer screening (combination of HFOBT and colorectal endoscopy). NHIS is a national survey that uses a multistage area probability design that permits representative sampling of households and noninstitutional group homes for face-to-face interview regardless of household telephone status. NHIS interviews a sample of approximately 30,000 to 40,000 US adults per year and achieves an annual response rate of approximately 80% of eligible households in the sample. Approximately three-quarters of US counties have no sample by survey design. BRFSS is a state-based system of health surveys administered by telephone. Since 2005, more than 350,000 adult interviews were completed each year, making BRFSS the largest telephone health survey in the world. The annual median state overall response rate for BRFSS is below 55%, which is typical of telephone surveys.

We used 2 external data sources to extract county-level ecological covariates for use in our small-area modeling. One was USA County Stats (7), which the US Census Bureau compiled from the 2000 and 2010 Census of Population and Housing (8), the American Community Survey (9), the Current Population Survey (10), the National Vital Statistics of the NCHS (11), and other administrative data sources. The other was the county-level variable file that CDC compiled in 2006 to supplement BRFSS; that file was created from data in the 2005 and 2006 Area Resource File, 2005 County Business Patterns, and Environmental Protection Agency Green Book Nonattainment Areas for Criteria Air Pollutants (12). We used the National Cancer Institute (NCI’s) SEER*Stat database (13) to extract county-level cancer mortality and incidence data (2008–2015) for external validation purposes.

### Outcomes

Outcomes were self-reported smoking and screening for female breast cancer, cervical cancer, and colorectal cancer derived from responses to the survey questions. To be included as an outcome, the survey questions had to be consistent with NHIS and BRFSS. We defined each outcome as follows:

**Current smoking:** Whether a person aged 18 or older reported currently smoking cigarettes some days or every day and having smoked at least 100 cigarettes in his or her lifetime by the time of interview. This category consists of 3 outcomes: current smokers overall, female current smokers, and male current smokers.

**Ever smoking:** Whether a person aged 18 or older smoked at least 100 cigarettes in his or her lifetime by the time of interview. This category includes 3 outcomes: ever smokers overall, female ever smokers, male ever smokers.

**Mammography screening for breast cancer:** whether a woman aged 40 or older had a mammogram within the 2 years preceding the interview.

**Papanicolaou screening for cervical cancer:** whether a woman aged 18 or older had a Papanicolaou test within the 3 years preceding the interview.

**HFOBT screening for colorectal cancer (CRC):** Whether a person aged 50 or older had an HFOBT within the 2 years preceding the interview.

**Colorectal endoscopy** **screening for CRC:** Whether a person aged 50 or older had at least 1 colorectal endoscopy (proctoscopy, sigmoidoscopy, or colonoscopy) at any time preceding the interview.

**Ever had a CRC screening**: Whether a person aged 50 or older had at least one HFOBT in the 2 years preceding the interview or at least 1 colorectal endoscopy at any time preceding the interview.

### Small area model and implementation

We considered area-level SAE models that first required us to compute county-level direct estimates of our outcomes of interest. Extending the models used in Raghunathan et al (6), we developed a hierarchical multilevel mixed effects model at the county level for each outcome.

NHIS samples were grouped into 3 exclusive groups based on household telephone status (households with a landline telephone, households with a cellular telephone only, and households without a telephone). For each outcome, national-level and county-level direct survey estimates of prevalence (eg, current smoking prevalence) were produced by using responses from BRFSS and NHIS, respectively. To incorporate the survey weights and complex sample design, we used the SAS PROC SURVEY package (SAS Institute) to produce these direct estimates for counties with responses from NHIS or BRFSS.

The first level of the small area model assumed an asymptotic distribution for the direct estimate vector of the 3 NHIS estimates by telephone status and BRFSS estimates. Arcsin-square-root transformations were applied to the direct estimates to stabilize the sampling variance (14). We used an unknown adjustment factor like the one used by Raghunathan et al (6) to measure the proportionate bias in BRFSS estimates relative to NHIS estimates.

The second level of the model incorporated a set of covariates and introduced random effects at the county level, which enabled borrowing of information among counties and induced smoothing. The covariates integrated from multiple alternative sources are given in Table A1 of the Appendix

Diffuse but proper prior assumptions were used for the hyperparameters. The Markov Chain Monte Carlo technique of Gibbs sampling (15) was adopted and implemented by using GAUSS programming software (Aptech Systems Inc) (Appendix).

### Model validation

The small area models assumed that for all outcomes of interest, BRFSS direct estimates — after dividing by the unknown adjustment factors — were unbiased estimates of the population means for the households with landline telephones Therefore, 1 model validation was to check the ratios of the BRFSS direct estimates (after adjusting for the difference between BRFSS and NHIS) to the model-based estimates for households with landline telephones. These ratios were expected to converge to one as the BRFSS county-level sample size increased.

We also computed the summary statistics (mean, standard deviation, minimum, and maximum) of the direct estimates and the modeled estimates by household telephone status across all of the counties, to detect outliers. In addition, we aggregated the county-level modeled estimates to the national level and compared those with the corresponding national-level direct estimates from both NHIS and BRFSS.

An external validation was also performed by linking the county-level smoking prevalence estimates to the most recent 5-year lung cancer mortality rate data (2011–2015), extracted from NCI’s SEER*Stat database (13), and by examining the relationship. We also linked cancer screening estimates with their corresponding cancer incidence (or mortality) rates and examined the relationship.

## Results

 We created county-level model-based estimates for the 11 outcomes for 3,112 counties in the United States. The remaining counties were excluded because some ecological covariates were not available. The final model-based SAEs of the outcomes were posted on NCI’s Small Area Estimates for Cancer-Related Measures website (https://sae.cancer.gov/nhis-brfss/) and included in NCI’s Surveillance, Epidemiology, and End Results (SEER) county attributes database (https://seer.cancer.gov/seerstat/variables/countyattribs/). The 2008–2010 estimates were also released via the State Cancer Profiles website (https://statecancerprofiles.cancer.gov/), which cancer control planners visit frequently. 

At the national-level, from 2008 through 2010, 75.0% of households had landline telephones, 23.2% of households had cellular telephones only, and 1.8% of households had no telephone (Table 1). Although households without telephones accounted for only a small percentage (1.8%) nationally, the percentage varied significantly across counties. For example, the county-level model-based estimates of the percentage of households without telephones varied from 0.2% to 18.1%, with a county mean of 2.0% across the 3,112 US counties included in this study. The county-level modeled estimates of percentage of cellular telephone-only households varied from 3.4% to 58.3%, with a county mean of 21.8%.

**Table 1:** National NHIS and BRFSS Direct Estimates of Prevalence for 11 Outcomes, by Household Telephone Status, 2008–2010^a^

Outcome	NHIS^b^				BRFSS, n = 1,276,268	Aggregated Modeled Small Area Estimates^c^
	Landline Telephone, n = 54,574	Cellular Telephone Only, n = 20,214	No Telephone, n = 1,753	Overall, n = 76,669		
Current smokers, all aged ≥18	54,157 (17.7) [0.2]	20,084 (27.3) [0.5]	1,731 (30.9) [1.4]	76,095 (20.2) [0.2]	1,269,021 (17.9) [0.1]	19.8
Current smokers, men ≥18	22,657 (19.9) [0.4]	9,843 (29.8) [0.6]	930 (35.0) [2.0]	33,488 (22.7) [0.3]	479,181 (20.0) [0.1]	22.1
Current smokers, women ≥18	31,500 (15.9) [0.3]	10,241 (24.6) [0.6]	801 (25.4) [1.9]	42,607 (17.8) [0.3]	789,840 (15.9) [0.1]	17.0
Ever smokers, all ≥18	54,176 (41.6) [0.3]	20,089 (43.2) [0.5]	1,731 (43.2) [1.5]	76,119 (42.0) [0.3]	1,269,021 (42.7) [0.1]	41.7
Ever smokers, men ≥18	22,664 (47.9) [0.4]	9,846 (47.7) [0.7]	930 (50.2) [2.1]	33,498 (47.9) [0.4]	479,181 (48.2) [0.2]	48.3
Ever smokers, women ≥18	31,512 (36.0) [0.4]	10,243 (38.2) [0.7]	801 (33.7) [2.1]	42,621 (36.4) [0.3]	789,840 (37.4) [0.1]	35.9
Mammography screening for breast cancer in past 2 years, women ≥40	13,821 (69.1) [0.5]	2,073 (56.6) [1.2]	219 (42.1) [3.6]	16,134 (67.4) [0.4]	417,325 (76.0) [0.1]	70.6
Papanicolaou test for cervical cancer in past 3 years, women ≥18	18,807 (73.1) [0.4]	6,132 (78.3) [0.7]	464 (68.8) [2.5]	25,435 (74.2) [0.4]	503,328 (77.9) [0.1]	76.1
HFOBT in past 2 years, all ≥50	17,056 (14.3) [0.3]	2,307 (12.5) [0.9]	352 (7.7) [2.0]	19,741 (14.0) [0.3]	523,726 (20.1) [0.1]	14.0
Ever had CRC endoscopy, all ≥50	17,587 (60.1) [0.5]	2,330 (45.4) [1.4]	350 (32.2) [3.1]	20,292 (58.3) [0.4]	531,018 (64.3) [0.1]	58.8
Ever had CRC screening, all ≥50	17,431 (64.5) [0.5]	2,316 (50.5) [1.4]	347 (35.5) [3.2]	20,119 (62.8) [0.4]	529,290 (69.4) [0.1]	63.5

Abbreviations: BRFSS, Behavioral Risk Factor Surveillance System; CRC, colorectal cancer; HFOBT, home fecal occult blood test; NHIS, National Health Interview Survey; SE, standard error.

a Values are number (percentage) [standard error] unless otherwise indicated.

b NHIS respondents with a landline telephone (75.0% of respondents), a cellular telephone (23.2% of respondents), or no telephone (1.8% of respondents). Values for 3 categories of telephone status do not equal overall total because some households were missing information on telephone status.

c Internal validation results, after aggregating the county-level modeled small area estimates to the national level.

NHIS direct estimates of the 11 outcomes varied by telephone status across households. The cellular telephone-only households and the households with no telephones typically had higher smoking rates and lower screening rates than the households with landline telephones. For example, the current smoking prevalence estimated from the NHIS was 17.7% in households with landline telephones, 27.3% in cellular telephone-only households, and 30.9% in households without telephones. The mammography screening rate among women aged 40 or older was 69.1% in households with landline telephones, 56.6% in cellular telephone-only households, and 42.1% in households without telephones. One exception was Papanicolaou screening where the cellular telephone-only households had the highest screening rates among the 3 household groups.

Comparing the NHIS and BRFSS estimates for the 11 outcomes, we noted that for prevalence of current smokers and ever smokers, BRFSS direct estimates (17.0%) and the NHIS direct estimates (17.7%) for households with landline telephones were almost identical; however, the BRFSS estimates were up to 2.7% lower compared with the NHIS overall estimates for some of the smoking outcomes. For cancer screening rates, BRFSS estimates were significantly higher than NHIS estimates for households with landline telephones (eg, 76.0% verse 69.1% for breast cancer screening). This is consistent with findings in the literature comparing the 2 surveys (6,16,17).

Table 2 provides the summary statistics and range (minimum, 25^th^ percentile, median, 75^th^ percentile, maximum, mean, standard deviation) of the county-level modeled estimates for the 11 outcomes across the 3,112 counties. The estimates for all 11 outcomes varied across the counties. The current county-level smoking prevalence in 2008–2010 varied from 6.8% (95% confidence interval [CI], 2.7%–11.0%) to 43.0% (95% CI, 26.6%–59.5%), with an average of 25.1% across the 3,112 counties. The prevalence of breast cancer screening within the last 2 years varied from 30.7% (95% CI, 17.9%–43.5%) to 94.7% (95% CI, 87.1%–100%). The prevalence of cervical cancer screening within the past 3 years varied from 42.8% (95% CI, 29.0%–56.5%) to 96.5% (95% CI, 91.5%–100%). The prevalence of ever having a colorectal endoscopy test or an HFOBT within the past 2 years varied from 27.8% (95% CI, 15.5%–40.0%) to 88.8% (95% CI, 79.0%–98.6%) across the 3,112 counties. The modeled estimates reduced the range, with a mean estimate that was closer to the NHIS estimate than the county-level NHIS and BRFSS direct estimates.

**Table 2:** Summary of County-Level Modeled Estimates of Cancer Risk Factors and Screening Behaviors for 11 Outcomes Across 3,112 US Counties, 2008–2010^a^

Outcome	Minimum	25th Percentile	Median	75th Percentile	Maximum	Mean	Standard Deviation
Current smokers, all aged ≥18	6.8	21.7	25.3	28.8	43.0	25.1	5.5
Current smokers, men ≥18	9.1	22.1	25.9	29.4	44.7	25.7	5.3
Current smokers, women ≥18	2.9	18.5	22.5	26.6	53.2	22.7	6.4
Ever smokers, all ≥18	22.0	44.4	49.0	53.0	73.7	48.6	6.8
Ever smokers, men ≥18	26.8	52.3	57.0	61.3	77.2	56.5	6.9
Ever smokers, women ≥18	15.3	36.7	42.4	47.1	73.6	41.9	7.8
Mammography screening for breast cancer in past 2 years, women ≥40	30.7	63.4	68.1	72.5	94.7	67.7	7.3
Papanicolaou test for cervical cancer in past 3 years, women ≥18	42.8	69.4	73.2	76.7	96.5	72.7	6.1
HFOBT in past 2 years, all ≥50	4.1	10.5	12.4	14.4	27.1	12.5	3.0
Ever had CRC endoscopy, all ≥50	26.8	52.0	56.5	60.7	88.0	56.1	7.0
Ever had CRC screening, all ≥50	27.8	56.7	61.4	65.4	88.8	60.7	7.2

Abbreviations: CRC, colorectal cancer; HFOBT, home fecal occult blood test; NHIS, National Health Interview Survey; BRFSS, Behavioral Risk Factor Surveillance System.

a Model-based estimates created by combining NHIS, BRFSS, and external auxiliary variables through novel statistical models. Values are percentages unless otherwise indicated.

The aggregated national modeled estimates for all 11 outcomes are similar to the corresponding NHIS national direct estimates, which is consistent with what we expected (Table 1).

**Figure 1:**
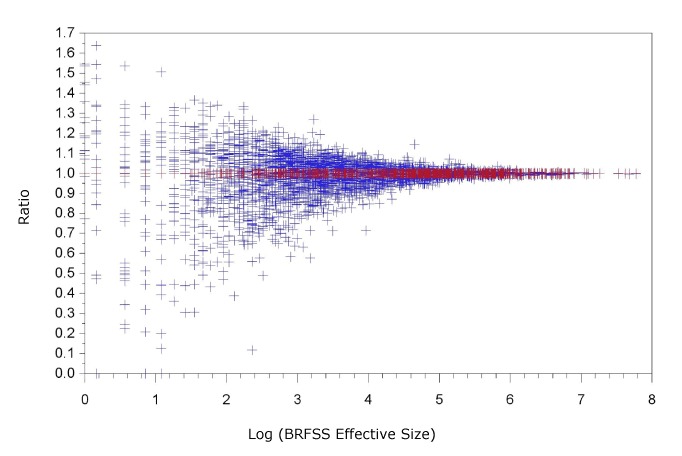
Ratio of Behavioral Risk Factor Surveillance System (BRFSS) direct estimates, after adjusting the difference between BRFSS and the National Health Interview Survey (NHIS), to the modeled estimates of breast cancer screening for households with landline telephones, 2008–2010.

 We plotted the ratios of the model-based estimates for households with landline telephones to the BRFSS direct estimates — after adjusting for the difference between the NHIS and BRFSS — against the BRFSS effective sample size (sample size divided by estimated design effect) on a log scale (Figure 1). The funnel shape indicates that the modeled estimates and the BRFSS difference-adjusted direct estimates match very well for large counties, as expected.

**Figure 2:**
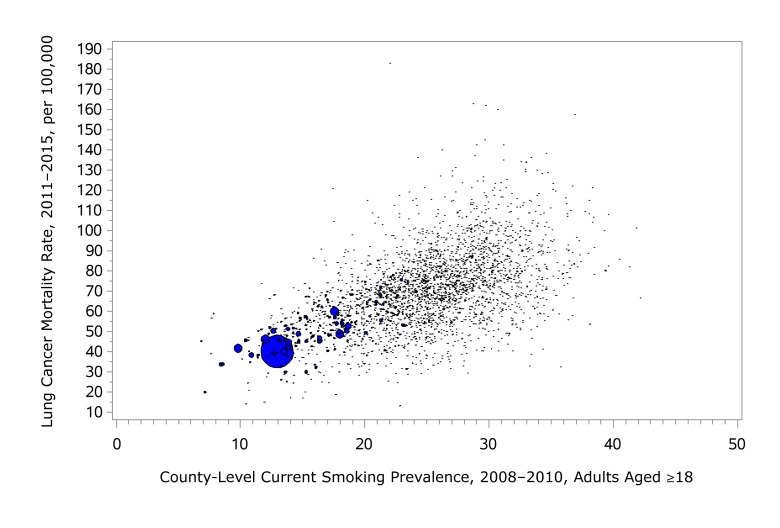
Bubble chart of county-level age-adjusted mortality rates (per 100,000) for 2011–2015 versus current smoking prevalence for 2008–2010 for adults aged 18 or older.

The weighted correlation coefficient between the 2008–2010 county-level modeled current smoking prevalence and the 2011–2015 age-adjusted county-level lung cancer mortality rate is 0.741 (*P* < .001), using the inverse variance of the lung cancer mortality rate as the weight. We calculated lung cancer mortality rates (2011–2015) against current smoking prevalence (2008–2010) in a bubble scatter plot, where the size of the bubble displays the inverse variance of the lung cancer mortality rate (Figure 2). Both the correlation coefficient and the scatter plot demonstrate a strong linear relationship between the modeled county-level current smoking prevalence and the county-level lung cancer mortality rates, even though they are only a few years apart. The correlation coefficient between county-level cancer screening and corresponding cancer mortality or incidence (eg, mammography and breast cancer) varies from cancer to cancer, but all are significant. This external validation is evidence that the SAE models perform well.

## Discussion

We generated county-level model-based estimates for 11 cancer risk factors and screening behaviors by combining information from NHIS, BRFSS, and auxiliary variables obtained from other data sources through novel statistical models for the data period 2008–2010. The same methods were used to produce SAEs for the data period 2004–2007. Our results revealed a large disparity in smoking prevalence and cancer screening rates among households by telephone status.

Our models have several strengths: 1) they use data from 2 large-scale national surveys, taking advantage of the large sample size from one (BRFSS) and the higher response rates and better coverage of all household types from the other (NHIS); 2) they incorporate cellular telephone–only households, a status that emerged rapidly during the study periods, as 1 dimension in the multivariate model structure, enabling better estimation; 3) they are built with county-level data, so survey weights and the major complex design features are incorporated before constructing the models; and 4) they include a large number of potential covariates, improving the predictive ability of the estimates. A limitation of the proposed methods is that we modeled the 11 outcomes separately and, to avoid further complicating the modeling process, didn’t consider the option of modeling all outcomes simultaneously. That approach may be worthy of exploration in future research. An additional limitation is that potential multicollinearity among the covariates may exist, thus possibly bringing potential bias to estimates of the regression coefficients. However, our main purpose was for prediction, not trying to interpret the relationship between the outcomes and the covariates.

Cancer screening is an important element of early detection and prevention (18). The US Preventive Services Task Force (USPSTF) makes recommendations on different types of cancer screening (19). Cancer screening metrics are included in the Healthy People 2020 goals (20), and the National Colorectal Cancer Roundtable aims to increase CRC screening prevalence to 80% by 2018 (21). However, cancer screening estimates for all US counties are not available elsewhere. Our SAEs are therefore an important and useful data resource for cancer control planners and researchers (22,23). Work has been initiated by the organizations responsible for these surveys, NCHS for NHIS and CDC for BRFSS, along with NCI, to analyze data from 2011 forward, in which a modified model will be developed to incorporate further changes in the BRFSS design, which now includes cellular telephone–only households and an improved weighting methodology. In addition, we encourage others to examine our methodology and develop other methodologies, to further examine the robustness of our results.

In defining the screening outcomes, we had to make some compromises between the latest USPSTF screening guidelines and the ability to code these outcomes consistently across time. For example, the addition of human papillomavirus (HPV) testing and immunization has changed the landscape of cervical cancer screening recommendations. In colorectal cancer screening, sigmoidoscopy is now rarely used in the United States, and newer technologies have been developed (eg, CT colonography, fecal DNA tests). We chose outcomes that, while not entirely current, could be coded consistently. These screening measures could serve as independent variables in other analyses or to judge areas of need. Although their estimates might not accurately reflect the newest screening technologies and guidelines, they are likely highly correlated and would likely maintain their rank order in counties across a state. In research using more recent data from 2011 forward, we tried to add estimates for cancer screening outcomes that align with the most recent USPSTF recommendations.

## Appendix

This appendix can be accessed at https://sae.cancer.gov/documents/appendix-sae-brfss.pdf.
